# Remodeling of the chromatin structure of the facioscapulohumeral muscular dystrophy (FSHD) locus and upregulation of FSHD-related gene 1 (*FRG1*) expression during human myogenic differentiation

**DOI:** 10.1186/1741-7007-7-41

**Published:** 2009-07-16

**Authors:** Beatrice Bodega, Gabriella Di Capua Ramirez, Florian Grasser, Stefania Cheli, Silvia Brunelli, Marina Mora, Raffaella Meneveri, Anna Marozzi, Stefan Mueller, Elena Battaglioli, Enrico Ginelli

**Affiliations:** 1 Department of Biology and Genetics for Medical Sciences, University of Milan, Milan, Italy; 2 Department of Biology II, Anthropology and Human Genetics, Ludwig Maximilians University, Munich, Germany; 3 Department of Experimental Medicine, University of Milan-Bicocca, Monza, Italy; 4 Neuromuscular Diseases and Neuroimmunology Unit, Muscle Cell Biology Laboratory, C. Besta Neurological Institute, Milan, Italy

## Abstract

**Background:**

Facioscapulohumeral muscular dystrophy (FSHD) is an autosomal dominant neuromuscular disorder associated with the partial deletion of integral numbers of 3.3 kb D4Z4 DNA repeats within the subtelomere of chromosome 4q. A number of candidate FSHD genes, adenine nucleotide translocator 1 gene (*ANT1*), FSHD-related gene 1 (*FRG1*), *FRG2 *and *DUX4c*, upstream of the D4Z4 array (FSHD locus), and double homeobox chromosome 4 (*DUX4*) within the repeat itself, are upregulated in some patients, thus suggesting an underlying perturbation of the chromatin structure. Furthermore, a mouse model overexpressing *FRG1 *has been generated, displaying skeletal muscle defects.

**Results:**

In the context of myogenic differentiation, we compared the chromatin structure and tridimensional interaction of the D4Z4 array and *FRG1 *gene promoter, and *FRG1 *expression, in control and FSHD cells. The *FRG1 *gene was prematurely expressed during FSHD myoblast differentiation, thus suggesting that the number of D4Z4 repeats in the array may affect the correct timing of *FRG1 *expression. Using chromosome conformation capture (3C) technology, we revealed that the *FRG1 *promoter and D4Z4 array physically interacted. Furthermore, this chromatin structure underwent dynamic changes during myogenic differentiation that led to the loosening of the *FRG1*/4q-D4Z4 array loop in myotubes. The *FRG1 *promoter in both normal and FSHD myoblasts was characterized by H3K27 trimethylation and Polycomb repressor complex binding, but these repression signs were replaced by H3K4 trimethylation during differentiation. The D4Z4 sequences behaved similarly, with H3K27 trimethylation and Polycomb binding being lost upon myogenic differentiation.

**Conclusion:**

We propose a model in which the D4Z4 array may play a critical chromatin function as an orchestrator of *in cis *chromatin loops, thus suggesting that this repeat may play a role in coordinating gene expression.

## Background

Facioscapulohumeral muscular dystrophy (FSHD) is an autosomal dominant disease with a prevalence of 1:20,000 [1] that is characterized by weakness and atrophy of the muscles of the face, upper arms and shoulder girdle. The FSHD locus has been mapped by linkage analysis to the subtelomeric region of the long arm of chromosome 4 (4q35) [2,3]. The disorder is associated with the deletion of an integral number of tandemly arrayed 3.3-kb units (D4Z4) [4]. Each D4Z4 repeat contains two homeoboxes within a single predicted open reading frame (ORF), double homeobox chromosome 4 (DUX4). The number of repeats varies from 11 to 110 in normal subjects, but is consistently less than 11 in FSHD patients [5], a contraction that is predominantly associated with a specific variant of chromosome 4 called 4qA [6-8]. Only a few patients with phenotypic FSHD show normal-sized D4Z4 repeats on both chromosomes 4. It is interesting to note that the D4Z4 repeats in patients with D4Z4 contractions or phenotypic FSHD show reduced levels of DNA methylation. D4Z4 hypomethylation is more prominent and present on both chromosomes 4 in patients with phenotypic FSHD, but it is restricted to the diseased chromosome in those with 4q-linked FSHD [9,10].

The region immediately proximal to the D4Z4 repeats harbors a number of candidate genes. This FSHD locus includes FSHD-related gene 1 (*FRG1*) [11], which encodes a nucleolar protein involved in RNA biogenesis [12]; *TUBB4q*, a member of the β-tubulin family; and *FRG2*, a predicted transcript with no significant homology to any known protein. The adenine nucleotide transporter 1 gene (*ANT1*), a gene involved in apoptosis, lies more distally from the 4qter (5.8 Mb) [13].

In addition to the DUX4 ORF within each unit of the tandem array, there is a DUX4-like sequence (named DUX4c) near *FRG2*. Preliminary data suggest that DUX4c may be expressed in FSHD samples [14], and it has been shown that its ectopic overexpression interferes with myogenic regulators and abolishes myoblast differentiation [15]. The expression of DUX4 RNA and protein has recently been selectively detected in primary myoblasts from FSHD patients, thus suggesting its involvement in FSHD [14,16]. Furthermore, the overexpression of DUX4 in different cell lines induces cell toxicity and apoptosis [14,17]. The overexpression of *FRG2*, *FRG1*, and *ANT1 *has been found in some muscles affected by FSHD [18-20]. It has also been shown that a transcriptional repressor complex binds D4Z4, and it is thought that D4Z4 deletion would trigger gene overexpression as a result of the lack of repression [18]; the overexpression of *FRG1*, but not *ANT1 *and *FRG2*, in transgenic mice leads to a general muscle dystrophy [21]. Furthermore, *FRG1 *overexpression in FSHD samples is not a uniform finding, [22,23] and thus the contribution of the *FRG1 *gene to the FSHD phenotype needs further validation. The transcriptional alterations reported above in some of the genes of the FSHD locus may be caused by a perturbation of the chromatin structure driven by the deletion of D4Z4 units [24].

This paper describes the crosstalking molecular events that occur within the FSHD locus. We concentrated on *FRG1 *because, although it is indicated as a candidate gene for FSHD pathogenesis, the molecular link between D4Z4 deletion and *FRG1 *deregulation remains unclear. We found that *FRG1 *is upregulated during myogenic differentiation and that FSHD myoblasts show significantly premature *FRG1 *expression in the early stages of differentiation. We investigated the chromatin features of *FRG1 *at hierarchical levels during myogenic differentiation in myoblasts derived from FSHD patients and normal individuals, and found that the *FRG1 *promoter undergoes chromatin remodeling involving the loss of the Polycomb repressor complex. Parallel analysis of D4Z4 repeats showed that the chromatin is marked by H3K27me3 (which is reduced in FSHD contracted alleles) and the Polycomb complex, and that both repressive markers are lost in myotubes. We further demonstrated that the physical interaction of the non-contiguous *FRG1 *and D4Z4 array, which is loosened in FSHD myoblasts, is remodeled upon cell differentiation. These results provide a connection between the deletion of D4Z4 repeats and the misregulation of the *FRG1 *candidate gene in FSHD cells.

## Results

### FRG1 expression is upregulated during the myogenic differentiation of human myoblasts

We investigated the mechanisms that regulate *FRG1 *gene transcription during the myogenic differentiation of human primary myoblasts. All experiments were performed using three myoblast cell lines derived from healthy donors, and four cell lines derived from FSHD patients (see Additional file 1a).

We first monitored myotube formation by means of immunostaining with sarcomeric myosin and MyoD antibodies after 2, 4 and 8 days of myogenic differentiation (Additional file 1b), and analyzed the expression of skeletal muscle-specific markers in myoblasts and myotubes after 8 days of differentiation (reverse transcription polymerase chain reaction (RT-PCR) analysis of myogenin, MyoD and myosin in Additional file 1c). The percentage formation was calculated as the number of 4',6-diamidino-2-phenylindole (DAPI)-positive nuclei in myotubes (positive cells) divided by the total number of nuclei in the area, and the obtained fusion index ranged from 60% to 90% (Additional file 1a).

The expression of sarcomeric myosin during myogenic differentiation (days 0, 1, 4 and 8) was checked by means of real time RT-PCR (Additional file 1d), and the results indicated that both the control and FSHD muscle cells were undergoing correct myogenic differentiation and had comparable differentiation properties.

**Figure 1:**
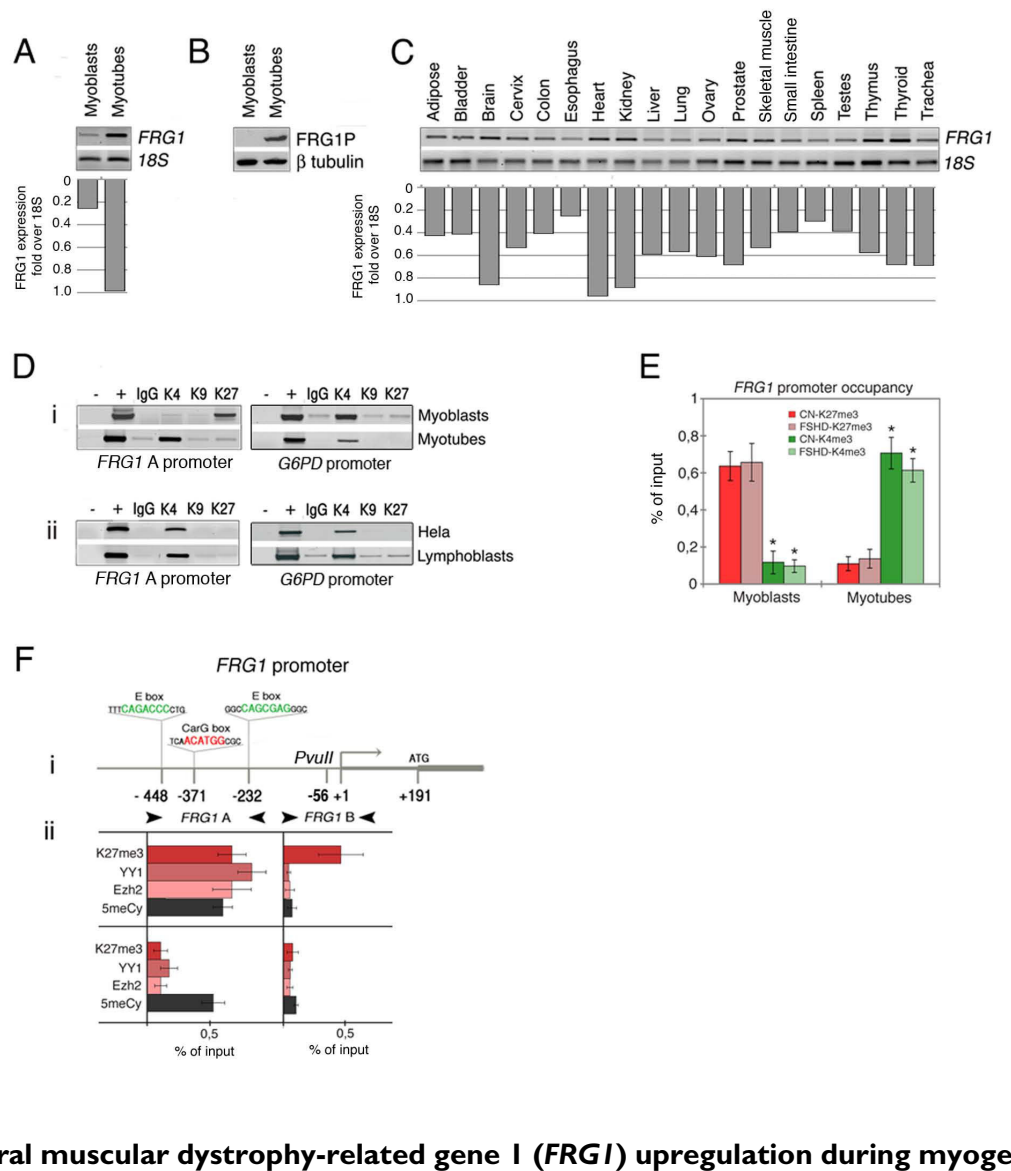
**Facioscapulohumeral muscular dystrophy-related gene 1 (*FRG1*) upregulation during myogenic differentiation is marked by a switch between H3K27me3 and Polycomb factors with H3K4me3 on its promoter**. *FRG1 *mRNA and protein were detected by reverse transcription polymerase chain reaction (RT-PCR) **(a) **and western blotting **(b) **in myoblasts and myotubes (8 days of differentiation); 18S rRNA and b tubulin were used as controls. **(c) **Total RNA from adult tissues was tested for *FRG1 *expression by means of RT-PCR; 18S rRNA was used as a control. Histograms in A and C represent *FRG1 *expression over 18S rRNA. **(d) **Chromatin immunoprecipitation (ChIP) assays of (i) myoblasts and myotubes, and (ii) HeLa and lymphoblasts, using antibodies against H3K4me3 (K4), H3K9me3 (K9) and H3K27me3 (K27). Input DNA (+) represents total chromatin, and IgG the immunoprecipitation by normal rabbit IgG. The amplified *FRG1 *promoter subregion corresponds to *FRG1 *A in **(f)**(i). The *G6PD *promoter was amplified as a negative H3K27me3 control. **(e) **ChIP analyses of control (CN) and facioscapulohumeral muscular dystrophy (FSHD) myoblasts and myotubes, indicating the standard error of the mean. A two-tailed t test was used for statistical analysis; the asterisks indicate the statistically significant differences at α = 0.05. CN-K27me3/CN-K4me3 in myoblasts: *P *= 0.0167, n = 3; FSHD-K27me3/FSHD-K4me3 in myoblasts: *P *= 0.0157, n = 4; CN-K27me3/CN-K4me3 in myotubes: *P *= 0.0006, n = 3; FSHD-K27me3/FSHD-K4me3 in myotubes: *P *< 0.0001, n = 4. The RT-PCR primer pairs were 4q specific [11], and are shown in Additional file 3; the anti-FRG1P antibody is specific for a 4q FRG1 peptide [21]. **(f)**(i) A schema of the *FRG1 *promoter showing the position of one CarG box responsive element (in red) and two E-boxes (in green) in relation to the ATG and transcription start site (+1), and the *Pvu*II site. The arrowheads indicate the primer positions for the *FRG1 A *and *FRG1 *B PCRs. (ii) ChIP and methylated DNA immunoprecipitation (MeDIP) experiments on myoblasts and myotubes using the anti-H3K27me3 (K27me3), anti-Ezh2, anti-YY1, and anti-5-methyl cytidine (5meCy) antibodies. All PCR experiments were performed in a linear range of amplification, and band intensities were measured using a Typhoon 9200 phosphoscanner and Image Quant analysis software; after subtracting the signals derived from IgG immunoprecipitation, the results were expressed as percentages of input DNA.

These experimental conditions were used to analyze *FRG1 *expression at transcriptional and protein levels by RT-PCR and western blotting. *FRG1 *mRNA and protein expression was higher in the myotubes than in the myoblasts (Figure 1a, b); these data were confirmed in all the available cell lines (data not shown). RT-PCR analysis of a panel of adult human tissue RNA showed that *FRG1 *expression was not restricted to muscle cells (Figure 1c). *FRG1 *therefore seems to be subject to muscle-specific regulation, but is not a classic muscle gene.

We then analyzed *FRG1 *myogenic regulation at the histone code level using chromatin immunoprecipitation (ChIP) experiments, and found that the H3K27 trimethylation (H3K27me3) repression marker (but not H3K9me3) on the *FRG1 *promoter in myoblasts was replaced by the H3K4me3 activation marker in myotubes (Figure 1d part i). H3K4me3 was also present on the *FRG1 *promoter in other human non-muscle cells expressing *FRG1 *[25], such as HeLa and lymphoblasts (Figure 1d part ii). These findings suggest that the very low *FRG1 *mRNA level observed in myoblasts is caused by active repression, based at least on the trimethylation of H3K27, whereas H3K4me3 is a hallmark of gene activation. The switch between H3K27me3 and H3K4me3 was monitored in all of the available myoblasts and myotubes, and found to be statistically significant in both controls and patients (Figure 1e).

It has been previously shown that several muscle-specific genes in myoblasts are silenced as a result of the promoter recruitment of the histone H3K27 methyltransferase (HKMT) Ezh2 via YY1, two key components of the Polycomb complex [26], and that changes in H3K27 and YY1 binding can be associated with DNA methylation [27]. Upon gene activation, YY1 and Ezh2 dissociate from their target promoters, H3K27 becomes hypomethylated, and MyoD and serum response factor (SRF) are recruited [26]. We analyzed the FRG1 promoter sequence using MatInspector software (Genomatix, Munich, Germany) and identified one CarG box (the DNA binding site of the YY1 protein) at position -371 from the transcriptional start site (+1), and two flanking E-boxes (the DNA binding site of the MyoD transcription factor) at positions -448 and -232 (Figure 1f part i). For these analyses, as the FRG1 genomic region is duplicated in the human genome [11,28], we designed primer pairs specifically aimed at the 4q FRG1 genomic copy and confirmed their selectivity by means of a panel of human somatic cell monohybrids (Additional file 2). We performed ChIP experiments on myoblasts and myotubes using antibodies against H3K27me3, YY1, Ezh2 targeting the FRG1 YY1 regulatory region (FRG1 A) and the proximal promoter (FRG1 B) as a control (Figure 1f part i) (see Additional file 3 for the PCR primer pairs). In myoblasts, YY1 and Ezh2 were detected on CarG box (FRG1 A), and H3K27me3 widely marked the FRG1 promoter (FRG1 A and FRG1 B) (Figure 1f part ii).

We also carried out a methylated DNA immunoprecipitation (MeDIP) assay of the same FRG1 promoter regions (FRG1 A and FRG1 B) to analyze the DNA methylation status of the promoter, and mapped a region of DNA methylation in correspondence with the YY1 binding site (compare FRG1 A and FRG1 B in Figure 1f part ii). After 8 days of differentiation, Polycomb complex binding and the H3K27me3 marker were lost (Figure 1f part ii); however, there was no change in the DNA methylation pattern of the promoter, as has been previously reported in the case of other muscle genes [29]. Additional file 4a shows examples of the ChIP and MeDIP analyses of the FRG1 promoter.

These results indicate that FRG1 gene expression is regulated during human myogenic differentiation, and that the gene behaves in the same way as other muscle fiber-specific genes [26].

### The FRG1 gene is prematurely expressed at early stages of FSHD myoblast differentiation

The finding of *FRG1 *upregulation in muscle biopsies of FSHD patients is controversial [16,18,21,23], and previous studies have failed to detect any significant difference of *FRG1 *expression levels in FSHD myoblasts [22,23,30]. We therefore compared the pattern of *FRG1 *mRNA expression during differentiation in FSHD and control cells.

**Figure 2:**
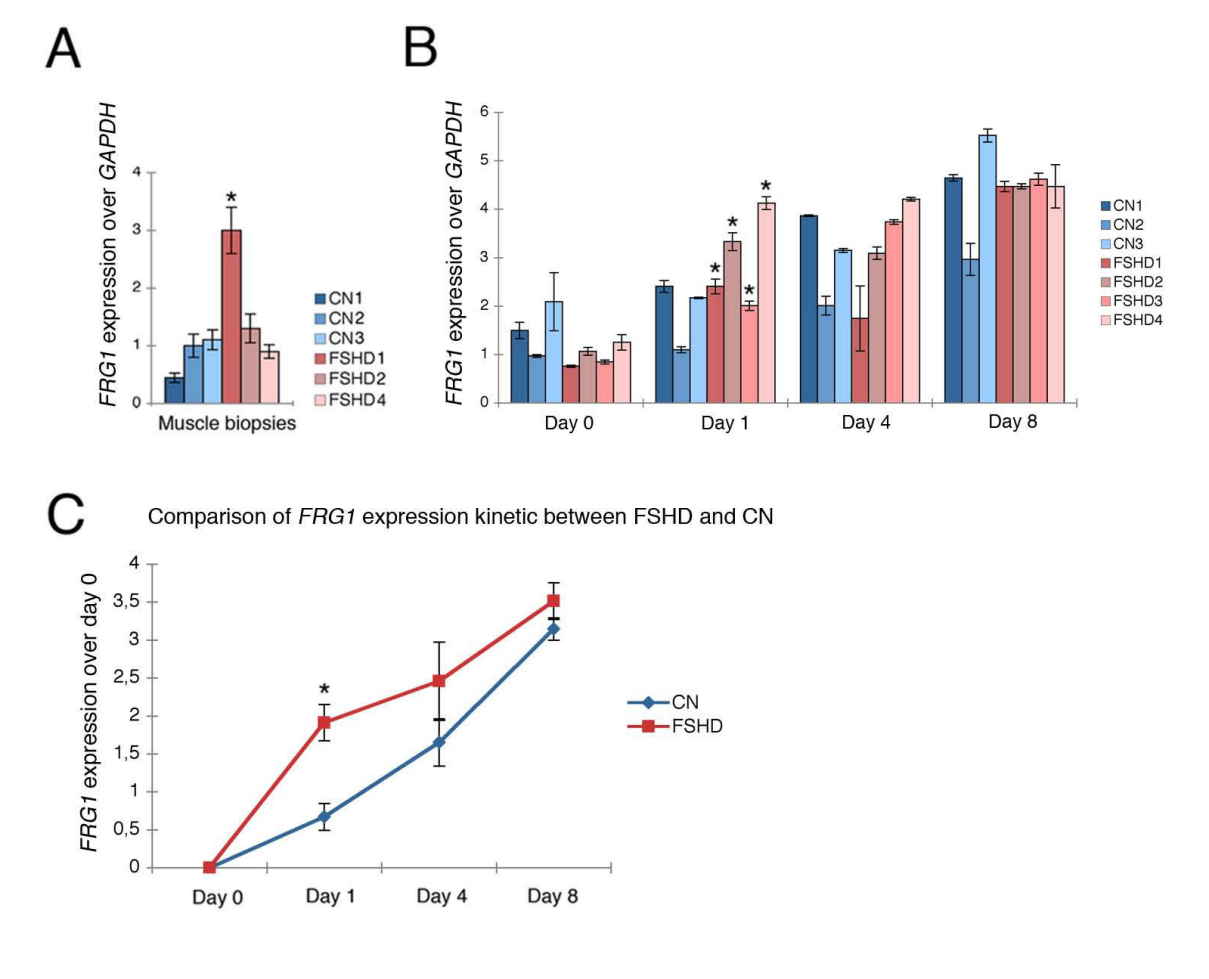
**Anticipated facioscapulohumeral muscular dystrophy-related gene 1 (*FRG1*) expression during facioscapulohumeral muscular dystrophy (FSHD) myoblast differentiation. (a) ***FRG1 *expression in three healthy (CN) and three FSHD muscle biopsies (FSHD) as revealed by means of a quantitative reverse transcription polymerase chain reaction (qRT-PCR) analysis relative to glyceraldehyde 3-phosphate dehydrogenase (GAPDH) expression. (see Additional file 3 for primers), indicating the standard deviation of the mean. A two-tailed t test was used for statistical analysis; the asterisk indicates the statistical significant difference between FSHD1 and CN3 (the control with the highest level of *FRG1 *expression): a = 0.05, *P *= 0.023. **(b) ***FRG1 *expression during myogenic differentiation in healthy (CN) and FSHD (FSHD) samples, expressed as fold of GAPDH expression, indicating the standard deviation of the mean. A two-tailed t test was used for statistical analysis; asterisks indicate the statistical significant differences between: FSHD1 day 1/FSHD1 day 0, α = 0.05, *P *= 0.0031; FSHD2 day 1/FSHD2 day 0, α = 0.05, *P *= 0.0035; FSHD3 day 1/FSHD3 day 0, α = 0.05, *P *= 0.0036; FSHD4 day 1/FSHD4 day 0, α = 0.05, *P *= 0.0033. **(c) **Kinetics of *FRG1 *expression during myogenic differentiation in control and FSHD cell lines; the values were determined as median of *FRG1 *expression at each step of differentiation (days 0, 1, 4 and 8) after subtracting the median value at day 0. The standard error of the mean was indicated. A two-tailed t test was used for statistical analysis; the asterisks indicate the statistically significant differences at α = 0.05; controls, n = 3; FSHD, n = 4; CN day 1/FSHD day 1, *P *= 0.0001. These results were derived from at least three independent RNA extractions for each human cell line.

*FRG1 *expression was analyzed in three FSHD and three control muscle biopsies by means of real time RT-PCR using primer pairs that exclusively amplified the 4q *FRG1 *copy [11]. In agreement with previous studies, we observed that *FRG1 *overexpression in muscle biopsies is not a uniform finding; indeed, only one FSHD sample showed *FRG1 *upregulation (Figure 2a). As *FRG1 *showed muscle-specific upregulation (Figure 1a), we performed this analysis in primary myoblasts derived from the same muscle biopsies and during their myogenic differentiation of (Figure 2b). No *FRG1 *overexpression was detected in FSHD myoblasts, as these samples did not show any statistical significant difference compared to control cells. Indeed, two control samples showed a basal level of *FRG1 *expression higher than FSHD. Moreover, all the samples showed *FRG1 *upregulation during myoblasts differentiation (from day 0 to day 8). In particular, the comparison of FSHD and control cells at each timepoint of differentiation for the increase of *FRG1 *mRNA level (in relation to the corresponding values at day 0) showed statistically significant results only for FSHD samples at day 1. We then derived the overall kinetics of *FRG1 *expression during myogenic differentiation of FSHD and control cells as median values of *FRG1 *expression at each point of differentiation (days 0, 1, 4 and 8), after the subtraction of the value at day 0 (Figure 2c). This analysis confirmed that in FSHD samples the *FRG1 *transcription at day 1 is significantly upregulated with respect to controls (Figure 2b, c). We verified that this was not due to a higher rate of differentiation because the myosin levels monitored at the same time were similar in the FSHD and control samples (Additional file 1d). In conclusion, these data suggest that the difference between patients and controls for the FRG1 expression states in the kinetics of transcription during myogenic differentiation.

### Chromatin structure and the nuclear topology of D4Z4 array in FSHD and control muscle cells

**Figure 3:**
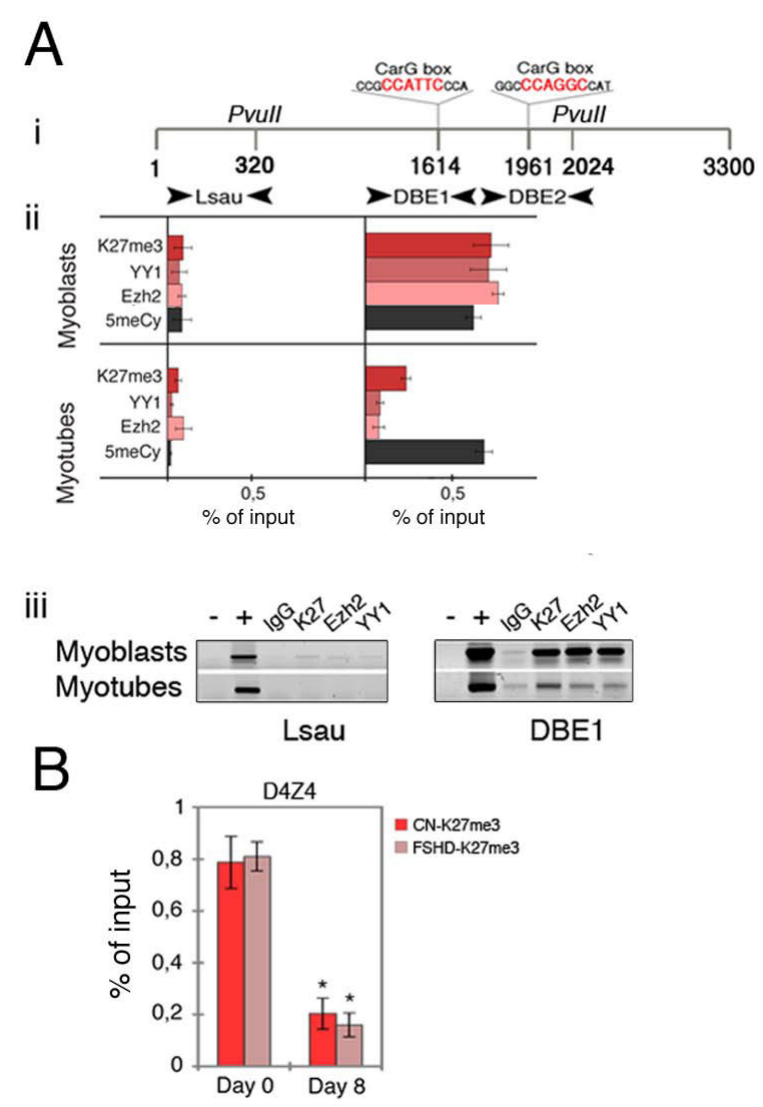
**Chromatin structure of D4Z4 units in human myoblasts**. **(a)**(i) A simplified schema of the D4Z4 unit showing the position of two CarG box responsive elements (sequences in red). The arrowheads indicate the primer positions for the Lsau, D4Z4 binding element (DBE)1 and DBE2 subregions; the *Pvu*II restriction site positions are indicated. (ii) Chromatin immunoprecipitation (ChIP) and methylated DNA immunoprecipitation (MeDIP) experiments on myoblasts and myotubes using anti-H3K27me3 (K27me3), Ezh2, YY1, and 5-methyl cytidine (5meCy) antibodies (iii) Examples of ChIP experiments on the Lsau and DBE1 subregions in myoblasts and myotubes. **(b) **H3K27 trimethylation of D4Z4 sequences before (day 0) and after (day 8) myogenic differentiation in healthy control (CN) and facioscapulohumeral muscular dystrophy (FSHD) cell lines, as revealed by ChIP experiments on the DBE1 subregion using anti-H3K27me3 antibody (red), indicating the standard error of the mean. A two-tailed t test was used for statistical analysis; the asterisks indicate the statistically significant differences at α = 0.05. CN-day 0/CN-day 8: P = 0.0172, n = 3; FSHD-day 0/FSHD-day 8: P = 0.0003, n = 4. All of the polymerase chain reaction (PCR) experiments were performed in a linear range of amplification, and band intensities were measured using a Typhoon 9200 phosphoscanner and Image Quant analysis software; after subtracting the signals derived from immunoprecipitation with IgG antibody, the results were expressed as percentages of input DNA. The primer pairs are shown in Additional file 3.

The same chromatin analyses as those described above were used to examine the D4Z4 sequences. The MatInspector software revealed two CarG boxes in specific subregions of the repeat, which are indicated as D4Z4 binding element (DBE)1 and DBE2 in the schema of Figure 3a part i. A YY1 binding site has been previously described in the D4Z4 repeat [18] and, in our schema, it coincides with the CarG box within the DBE1 region (Figure 3a part i).

We then performed ChIP experiments on three 3.3 kb subregions of the D4Z4 unit (Lsau, DBE1 and DBE2) (Figure 3a part i). The results obtained for DBE1 and Lsau are summarized in Figure 3a part ii; the results for DBE2 (not shown) were the same as those obtained for DBE1. In myoblasts, the YY1 and Ezh2 Polycomb factors and H3K27me3 were associated with the CarG box-containing sequences (DBE1 and DBE2), but not with Lsau; in myotubes, the Polycomb factors were no longer present in any of the regions (Figure 3a part ii). Furthermore, as with the FRG1 promoter (Figure 1f part ii), only YY1 binding sites were methylated, regardless of the presence of YY1 binding (before and after myogenic differentiation). Examples of the ChIP analyses of the D4Z4 subregions are shown in Figure 3a part iii.

To test the hypothesis that the premature activation of the FRG1 gene during the differentiation of FSHD cells may have been due to a weaker repression mechanism mediated by the contracted D4Z4 allele, we compared H3K27 trimethylation levels in the D4Z4 repeats in control and FSHD myoblasts before and after myogenic differentiation. As shown in Figure 3b, the ChIP experiments did not detect any appreciable differences in the H3K27 trimethylation between the healthy controls and FSHD cells. Because of the extensive duplication of D4Z4 sequences in the human genome [25,31], the PCR primers used were not 4q specific (see Additional file 2), and so the results refer to all of the D4Z4 sequences in the nucleus. It is therefore possible that a difference in D4Z4 H3K27 methylation between normal and FSHD cells (attributable to the fewer D4Z4 repeats in the contracted allele) may lead to a variation that is too small to be detected by the ChIP assay. However, ChIP experiments using monochromosomal cell hybrids retaining chromosome 4 indicate the presence of H3K27 trimethylation and YY1 on DBE sequences (D. Cabianca and D. Gabellini, personal communication, Division of Regenerative Medicine San Raffaele Scientific Institute DIBIT, Milan, Italy); moreover, MeDIP DNA methylation experiments using monochromosomal cell hybrids retaining chromosomes 4, 10 and acrocentrics show that only the DBE sequences on chromosome 4 and 10 are methylated (Additional file 4b).

Furthermore, myogenic differentiation triggered the chromatin remodeling of the D4Z4 sequences as a whole and induced a significant reduction in the Polycomb signals (H3K27me3, YY1 and Ezh2) (Figure 3a part iii). Taken together these results suggest that 4q D4Z4 repeats may be represented in samples immunoprecipitated by H3K27me3, YY1 and Ezh2 antibodies. However, as the primer pairs used amplify DBE sequences from many chromosomes (see Additional file 2), the obtained ChIP signals might also derive from genomic localizations of D4Z4 other than 4q. Since ChIP experiments do not provide direct evidence on the chromatin structure of the 4q D4Z4 array in myoblasts or on the presence of differences between healthy and FSHD cells, we used 3D fluorescence *in situ *hybridization (3D-FISH) as a complementary strategy.

**Figure 4:**
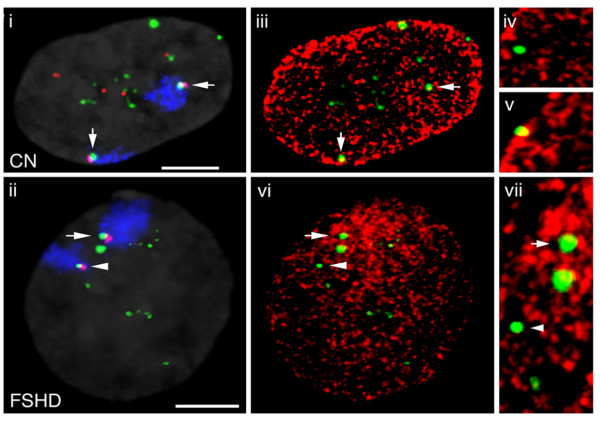
**Nuclear topology of D4Z4 units in control and FSHD myoblasts**. 4q subtelomere architecture in 1.4 μm midprojections of 3D-preserved interphase nuclei from healthy (i, iii, iv and v) and FSHD myoblasts (ii, vi and vii) immunofluorescence in situ hybridization (immuno-FISH) using anti-H3K27me3 antibody (scale bar = 5 μm). The chromosome 4q territories are shown in blue, the bacterial artificial chromosome (BAC) upstream of the FRG1 gene in red, and the BAC containing a D4Z4 array in green. The arrows highlight the 4q subtelomeres identified by the cohybridization of both BACs and the chromosome 4 painting. The arrowhead in (ii) identifies a 4q subtelomere showing reduced hybridization with the green BAC and probably corresponding to the contracted D4Z4 allele. (iii) and (vi) The same nuclei as those respectively shown in (i) and (ii) were immunostained with anti-H3K27me3 antibody (red); the green spots correspond to hybridization with the D4Z4-containing BAC; the arrows and arrowhead identify the 4q alleles. (iv) and (v) Representative confocal sections of a nucleus from healthy myoblasts consisting of 4q D4Z4 alleles (green) that were negative ((iv), only green) or positive ((v), green and yellow) for colocalization with anti-H3K27me3 immunofluorescence (red). (vii) A 3 × enlargement of the confocal section in (vi) showing a nucleus from FSHD myoblast consisting of 4q D4Z4 alleles (green) that were negative (arrowhead, only green) or positive (arrow, green and yellow) for colocalization with anti-H3K27me3 immunofluorescence (red).

To identify the 4q subtelomere, we used a cocktail of three DNA probes: two were bacterial artificial chromosomes (BACs) of the FSHD locus, one containing D4Z4 repeats (green in Figure 4 part i) and one not overlapping located 130 kb upstream of the D4Z4 array (red in Figure 4 parts i and ii) (see Methods for details), and the third was a painting for chromosome 4 territory (blue in Figure 4 parts i and ii). We followed their colocalization in order to analyze the nuclear architecture of the FSHD locus (arrows in Figure 4) and distinguish the two 4q alleles in the nucleus; one 4q allele in almost all FSHD nuclei shows a weaker hybridization signal [32], and this was considered the bona fide contracted allele (Figure 4 part ii, arrowheads). We then investigated the nuclear position of the FSHD locus identified by the three probes (relative to dense H3K27me3 signals) in FSHD and control myoblast nuclei.

The nuclei were stained with an H3K27me3-specific antibody (red in Figure 4 parts iii, iv, v, vi and vii), and 30 were scored in both the healthy and FSHD myoblasts. Colocalization with anti-H3K27me3 immunofluorescence (yellow in Figure 4 part iii) was observed in 72.4% of the 4q alleles in the healthy cells (examples of negative and positive H3K27me3 colocalization are shown in Figure 4 parts iv and v). In particular, the majority of the non-4q D4Z4 signals (mainly attributed to the short arms of acrocentric chromosomes) [25,31] were concentrated in nucleolar subdomains that are known to be H3K27me3 negative [33]. In contrast, the FSHD nuclei showed colocalization with H3K27me3 signals in only 38.6% of the 4q alleles (yellow in Figure 4 parts vi and vii) (P = 0.0013). The H3K27me3 colocalized signals were attributed mainly to the wild-type allele identified by the larger hybridization spot (Figure 4 part ii). The observed reduction in the colocalization of H3K27me3 signals and contracted alleles may have been due to the fact that the FSHD alleles give smaller FISH spots, thus preventing us from concluding that the contracted alleles were H3K27 hypomethylated; nevertheless, the different size of the spot undoubtedly reflects a different concentration of H3K27me3 in that subregion of the FSHD nucleus. This experiment, which allows the direct visualization of the tridimensional chromatin structure in vivo, revealed that the majority of D4Z4 sequences modified by H3K27me3 belonged to the array on chromosome 4; moreover, we detected significant differences between the patients and controls that cannot be detected by high resolution PCR-based techniques such as ChIP. Paradoxically, when working with highly repeated DNA sequences clustered on several chromosomes, it is essential to use an approach that distinguishes the genomic derivations of the signals in order to be able to monitor differences attributable to a specific chromosomal locus (that is to say, the D4Z4 array on chromosome 4q) even with a low-resolution technique.

### A specific 4q D4Z4-mediated chromatin loop involving the FRG1 promoter is dynamically regulated during human myoblast differentiation

**Figure 5:**
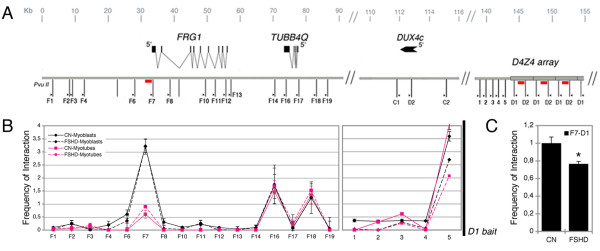
**Higher order structure of the facioscapulohumeral muscular dystrophy (FSHD) locus**. **(a) **Diagram of the genomic region analyzed in the chromosome conformation capture (3C) experiments, indicating the *Pvu*II restriction sites (thin vertical lines); the arrowheads indicate the primer positions, the F series in FSHD-related gene 1(*FRG1*), the C series in DUX4c, the D series in the D4Z4 repeats; the numbers from 1 to 5 indicate the restriction sites near the D1 bait used as positive controls. The red rectangles show the locations of CarG boxes. **(b) **The Y axis represents the crosslinking frequency expressed as the ratio of polymerase chain reaction (PCR) performed on 3C samples relative to bacterial artificial chromosome (BAC) controls between the fixed *Pvu*II fragment D1 (D4Z4 repeats) and the rest of the FSHD locus after the correction for digestion and ligation. The calculation of the relative crosslinking frequency between two given fragments, performed as described previously [43], allows a direct comparison between the different cell types used in the 3C assay by correcting for possible variants. All data points were generated from an average of three independent experiments. The standard error of the mean is indicated. One-way analysis of variance (ANOVA) was applied for statistical analysis; control myoblasts: α = 0.05, *P *= 1.81*10^-12^; control myotubes: α = 0.05, *P *= 3.14*10^-6^. To compare myoblasts and myotubes, two-way ANOVA was applied for statistical analysis: α = 0.05, *P *= 0.0073. **(c) **Frequency of interaction between the *FRG1 *promoter and D4Z4 sequences in FSHD and control myoblasts, indicating the standard error of the mean. Quantitative (q)PCR was performed on 3C templates using a TaqMan probe complementary to the *FRG1 *promoter portion of the PCR product obtained using F7 and D1 primers. The results are normalized to digestion, ligation and crosslinking efficiency, as described in Additional file 5. A two-tailed t test was used for statistical analysis; the asterisks indicate the statistically significant differences at α = 0.05. Controls, n = 3; FSHD, n = 4; *P *= 0.021. The primers are shown in the detailed 3C protocol section of Additional file 5.

It has been recently demonstrated that the formation of higher order structures is a powerful mechanism coordinating the expression of distant loci in the genome [34]. In order to verify whether the D4Z4 array and *FRG1 *gene physically interact in human myoblasts and myotubes, we investigated this association using chromosome conformation capture (3C) technology [35] (see Additional file 5 for the detailed protocol), and scanned 90 kb spanning the FSHD locus using primers flanking *Pvu*II restriction sites (Figure 5a). The *Pvu*II restriction enzyme was selected because it cuts in proximity to CarG sequences on both *FRG1 *and the D4Z4 unit (see Figure 1f part i and Figure 3a part i).

A number of controls are essential for the correct interpretation of 3C data [36]. We confirmed that all of the 3C primers amplified artificial 3C products *in vitro *(see Additional file 6a and Additional file 5 for details), and did not amplify undigested and ligated, or digested but not ligated, chromatin (data not shown). Furthermore, all of the 3C products derived from crosslinked and BAC templates were sequenced and corresponded to the expected sequences (not shown). We selected two baits in the proximity of the two *Pvu*II restriction sites in the D4Z4 unit: one (D1) detected the *Pvu*II-D4Z4 fragment containing the CarG sequences and discriminated D4Z4 repeats and the unique copy located between the *FRG1 *and *FRG2 *genes (DUX4c); the second (D2) detected the remaining portion of the repeat and also a DUX4c *Pvu*II fragment (see the schema in Figure 5a). We also designed two baits specific for DUX4c *Pvu*II fragments (C1 and C2). A total of 16 preys (F series) were positioned flanking the *Pvu*II sites in the *FRG1 *genomic region (see schema in Figure 5a), and an additional 5 proximal to the D1 baits (1 to 5 in Figure 5a) were used to confirm that interactions between D1 and the nearby *Pvu*II restriction fragments were quantitatively detected (an index validating the quality of a 3C assay). We measured the crosslinking frequencies between these baits and the various *Pvu*II sites throughout the FSHD locus. D1 (Figure 5b) and D2 (not shown) showed one intrachromosomal loop between the D4Z4 array and the *FRG1 *promoter (F7) in human myoblasts, and two additional interactions with the TUBB4q gene (F16 and F18). Interestingly, the D4Z4/*FRG1 *promoter interaction was specific to the *Pvu*II fragment-containing CarG box (red rectangle in Figure 5a). Additional file 6a shows an example of these findings.

To test the correlation between the derived chromatin structure and the transcriptional potential of the *FRG1 *promoter, we performed similar 3C analyses after myogenic differentiation. In striking contrast to the presence of long-range associations between D4Z4, *FRG1 *and the *TUBB4q *in undifferentiated myoblasts, myogenic differentiation led to an almost complete loss of the interaction with the *FRG1 *promoter (*P *< 0.05 in all cases, see Additional file 6b, c for examples), and the locus acquired a more relaxed conformation; conversely, the interaction between D4Z4 and *TUBB4q *did not seem to be affected by the differentiation process (Figure 5b). The same 3C analysis of a monochromosomal cell hybrid retaining human chromosome 4 gave similar results (Additional file 7b), thus demonstrating that the loops were formed between sequence elements localized on chromosome 4.

To investigate whether DUX4c could also mediate similar interactions, we set up a 3C analysis using the C1 and C2 baits described above. The use of C1 revealed an interaction between the *FRG1 *promoter and the DUX4c fragment that did not change during differentiation (Additional file 7c), whereas no interactions were revealed by C2 (not shown). Notably, the relative crosslinking frequency was one order of magnitude lower with the DUX4c bait than with the D4Z4 baits (compare F7-D1 with F7-C1 in Figure 5b, and Additional file 7c).

As the expression of the *FRG1 *gene is misregulated in FSHD cells (Figure 2), we investigated whether the D4Z4 contraction affects loop formation by making the same 3C analysis of FSHD myoblasts and myotubes, but we did not find any significant differences from controls (Figure 5b). However, reasoning that the wild-type allele may mask a possible small reduction in the frequency of the interactions, we used a 3C quantitative PCR (3C-qPCR) assay in order to determine the crosslinking frequencies of the F7-D1 interaction more accurately [37]. We designed a TaqMan probe complementary to the *FRG1 *promoter of the hybrid fragment generated by 3C, and normalized the 3C-qPCR results following the same procedures as those used for the other 3C-PCRs (that is to say, normalization by digestion, ligation and crosslinking efficiency). This time, there was a slight but statistical significant reduction in the interaction frequency of the FSHD myoblasts (Figure 5c). However, it is possible that a difference in the D4Z4-*FRG1 *interaction between normal and FSHD cells (attributable to the fewer D4Z4 repeats in the contracted allele) may lead to a variation that is too small to be detected by the 3C assay. These findings suggest that the FSHD locus in muscle cells is structured into at least three intrachromosomal loops: two steady loops connecting D4Z4-*TUBB4q*, and one dynamic loop between D4Z4 and the *FRG1 *promoter that is relaxed during myogenic differentiation. This indicates that the higher order structure of these elements undergoes dynamic remodeling, which probably correlates with the disappearance of the Polycomb complex from both the *FRG1 *promoter and the D4Z4 array, and is concomitant with the upregulation of gene expression. Moreover, the ability to form loops between the *FRG1 *promoter and the D4Z4 array seems to be less efficient in FSHD myoblasts than in controls.

## Discussion

Facioscapulohumeral dystrophy is considered an epigenetic disorder [24]. Abnormalities in the expression of candidate genes such as *ANT1*, *FRG1 *and *FRG2*, and in the transcription of the D4Z4 repeat, have been reported in FSHD patients [24], but the chromatin features of the FSHD locus have not been studied in detail.

We found that the upregulation of *FRG1 *in FSHD patients is a gain of function mechanism that could explain the autosomal dominant inheritance of the disease, and that it is revealed only when myogenic differentiation triggers the remodeling of the locus. FRG1P is a nuclear protein that is thought to be involved in RNA processing [12,38]. Slight differences in the cell levels of regulatory proteins such as FRG1P may affect a number of factors and have multiple effects on cell physiology. For instance, the alternative splicing of muscle-specific genes is abnormally regulated in mice overexpressing *FRG1 *and showing an FSHD phenotype [21], and so inappropriate regulation of *FRG1 *during the early phases of muscle differentiation may have serious effects on the formation of muscle fiber. We suggest that *FRG1 *misregulation in a specific window of muscle differentiation may contribute to FSHD, although it cannot be considered the only molecular defect causing the FSHD phenotype: for example, the transcription of DUX4 recently observed in FSHD myoblasts [14,16] may contribute to the manifestation of FSHD.

In order to investigate the molecular basis of the *FRG1 *transcriptional alteration, we made a detailed analysis of the chromatin structure of two DNA regions residing in the FSHD locus in a human model of myogenic differentiation: the candidate gene *FRG1 *and the D4Z4 array, to which the genetic mutation underlying the disease has been mapped. These two DNA regions were studied at different levels of the epigenome, from DNA methylation and histone code modifications to higher order structures. In this regard, it is important to point out the intrinsic limitation of molecularly analyzing repetitive DNA elements, and so we used the complementary approaches of ChIP and 3D-FISH analysis to gain insights into the chromatin structure of D4Z4.

The analyses showed that the FSHD locus undergoes chromatin remodeling during myogenic differentiation. In normal myoblasts, the *FRG1 *gene is repressed and its promoter physically interacts with the D4Z4 array; upon differentiation, the Polycomb complex dissociates from the *FRG1 *promoter and the *FRG1 *gene is expressed. Like the *FRG1 *promoter, D4Z4 chromatin also shows the presence of the Polycomb complex and H3K27me3 in myoblasts, and their loss in myotubes; moreover, D4Z4 and the *FRG1 *promoter physically interact in myoblasts, whereas this chromatin loop is relaxed upon myogenic differentiation.

These data support the hypothesis that the 4q D4Z4 array may have a regulatory effect on *FRG1 *expression, which we suggest is due to their physical association in the nucleus. It has recently been demonstrated that Polycomb occupancy can repress transcription by maintaining a series of long-range chromatin interactions that are lost when mammalian cells differentiate [39], and so it would be interesting to investigate directly the involvement of the Polycomb repressor complex as a mediator of the *FRG1*-D4Z4 chromatin loop in myoblasts.

Chromatin characterization of *FRG1 *and the D4Z4 array in FSHD myoblasts revealed a reduction in H3K27me3 on the contracted D4Z4 allele, and a kinetic analysis of Polycomb dissociation during differentiation that was very similar to that observed in the control cells. The reduction in H3K27me3 may be due to the decrease in the number of D4Z4 units or to hypomethylation of the residual repeats. Furthermore, the early expression of *FRG1 *in differentiating FSHD myoblasts may indicate that muscle cells, like their non-muscle counterparts, require the recruitment of additional factors in order to activate *FRG1 *expression.

In our cell system, the regulation of *FRG1 *expression therefore seems to be preferentially conditioned by the chromatin structure of the region (that is to say, the strength of the loop between the *FRG1 *promoter and the D4Z4 array related to its chromatin structure). We found a slight reduction in the frequency of loop formation between the D4Z4 array and the *FRG1 *promoter in FSHD myoblasts in comparison with control cells. D4Z4 contraction in FSHD cells may qualitatively alter the repressive effect of this chromatin loop affecting the correct timing of *FRG1 *expression. It is possible that relaxed looping in the presence of protein factors that may induce further changes in chromatin conformation and/or more efficient transcription allows the expression of the *FRG1 *gene. Nonetheless, the observed reduction in the frequency of loop formation between the D4Z4 array and the *FRG1 *promoter in FSHD myoblasts is too small to certainly infer its involvement in the misregulation of *FRG1 *gene expression, and thus further experiments are required to link macrosatellite contraction and gene expression.

Pirozhokova *et al*. [40] published a 3C analysis of the FSHD locus and described the formation of loops between DUX4c and the *FRG1 *promoter. We detected the same loop in myoblasts, although the frequency of the interaction was one order of magnitude lower than that of the loop between *FRG1 *and D4Z4 sequences. The same authors also found a second loop between a telomeric element downstream of the D4Z4 array (the 4qA/B marker) and the *FRG1 *promoter only in FSHD myoblasts, and suggested that this element may enhance the transcription of the gene [40]. As we did not detect *FRG1 *up regulation in FSHD myoblasts, we suggest that the interaction with the 4qA/B marker have the proposed effect of transcription enhancement on *FRG1 *expression only when myogenic differentiation is triggered. Our data, together with data of Petrov *et al*. and Pirozhokova *et al*. indicate that the tridimensional structure of the FSHD region is functional for the expression of the *FRG*1 gene, and probably more than one sequence elements (for example, D4Z4, DUX4c, MAR region) could contribute to the fine regulation of gene expression [40,41].

Finally, our model may also explain the manifestation of FSHD in the absence of D4Z4 contraction, as in the case of phenotypic FSHD in which a wild-type 4q D4Z4 array is strongly hypomethylated [9,10]. In this case, D4Z4 hypomethylation may impair the Polycomb recruitment that leads to a reduction in H3K27 trimethylation, the same molecular defect that we observed in contracted 4q alleles.

## Conclusion

The results of this study provide the first demonstration of a mechanistic link between the D4Z4 array and the transcriptional regulation of muscle-specific *FRG1 *via intrachromosomal looping. This finding may predict a broader role of the D4Z4 array in coordinating regulatory interactions between non-contiguous elements in the genome. The identification of such *in cis *or *in trans *interactions may untangle the intricate cascade of events underlying the manifestation of FSHD and may suggest that this human disorder is an example of the structural modification of the epigenome.

## Methods

### Cell cultures, immunofluorescence

Human primary myoblasts from healthy donors and FSHD patients were obtained from the Telethon BioBank, (Neuromuscular Diseases and Neuroimmunology Unit, Muscle Cell Biology Laboratory, C. Besta Neurological Institute). The cell lines were cultured in Dulbecco's modified Eagle medium (DMEM) supplemented with 20% fetal bovine serum (FBS), insulin 10 mg/ml, human fibroblast growth factor (hFGF) 25 ng/ml, human epidermal growth factor (hEGF) 10 ng/ml (proliferating medium), and then induced to differentiate by means of DMEM supplemented with 2% horse serum (differentiating medium). All of the patients satisfied the accepted clinical criteria for FSHD. They had undergone DNA diagnosis and were identified as carriers of small (<38 kb, <11 repeats) 4q35-located D4Z4 repeat arrays, as determined by p13E-11 hybridization to *Eco*RI-digested and *Eco*RI/*Bln*I-digested genomic DNA. The details of the cell lines are shown in Additional file 2. The HeLa and human lymphoblasts were cultured in DMEM and RPMI supplemented with 10% FBS, whereas the monochromosomal somatic cell hybrids retaining human chromosome 4, 10, 15 and 22 (a gift of Professor M. Rocchi, Department of Genetics and Microbiology, University of Bari, Italy) were cultured in DMEM supplemented with 10% FBS. Cell immunofluorescence was performed using the antibodies MF20 1:3 (DSHB, University of Iowa, Iowa City, Iowa) and anti-MyoD 1:100 (Dako, Dako Italia, Milan, Italy).

### Total RNA extraction, RT-PCR and quantitative RT-PCR analysis

Total RNA was isolated from the cells using the RNeasy Mini Kit (Qiagen, Milan, Italy), and the purified RNA was treated with RNase-free DNase (Qiagen, Milan, Italy) to remove any residual DNA.

Quantitative RT-PCR (qRT-PCR) analysis was performed on an iQ5 real time PCR detection system (BioRad, Segrate, Italy) using the iScript two-step RT-PCR Kit with SYBR Green (BioRad, Segrate, Italy). The relative expression of the investigated genes was quantified after normalization against β_2 _microglobulin, the 18S subunit of ribosomal RNA and glyceraldehyde 3-phosphate dehydrogenase (GAPDH). For FRG1 and GAPDH real time RT-PCR correlation coefficient for the amplification and PCR efficiency were respectively R^2 ^= 0.963 and R^2 ^= 0.990, and 99.4% and 99.8%.

The primer pairs used for the real time amplifications are shown in Additional file 3.

### Protein isolation and western blotting

Total proteins were extracted using radioimmunoprecipitation assay (RIPA) buffer and the western blot analysis was carried out using standard techniques and the antibodies anti-histone H3 (AbCam, Cambrige, UK) and FRGIP (kindly provided by D. Gabellini, Division of Regenerative Medicine San Raffaele Scientific Institute DIBIT, Milan, Italy); polyclonal anti-β tubulin (Sigma, Milan, Italy) was used as standard.

### ChIP and MeDIP assays

ChIP was performed using 5 μg of normal rabbit IgG or antibodies against Ezh2 (Zymed, South San Francisco, California, US), and YY1 (Santa Cruz Biotechnology, Heidelberg, Germany), or antibodies against trimethylated H3K4 (Upstate Biotech, Millipore, Milan, Italy), trimethylated H3K9 (Upstate Biotech, Millipore, Milan, Italy), and trimethylated H3K27 (Upstate Biotech, Millipore, Milan, Italy).

For the MeDIP assay, 5 μg of sonicated genomic DNA (size: 200 to 600 bp) were immunoprecipitated with 5 vg of antibody against 5-methyl cytidine (Diagenode Biosence, Milan, Italy), and the immunoprecipitates were collected for 3 h at 4°C with constant agitation using 10 μl tRNA (20 mg/ml), 20 μl salmon sperm DNA (10 mg/ml) and 20 μl protein A-agarose beads added to the 1 ml samples.

### Cell fixation and immuno-FISH pretreatment

Healthy and FSHD myoblast cell lines were grown on cover slips for 12 to 24 h, and the cells were fixed in 4% paraformaldehyde (PFA) in 1 × phosphate buffered saline (PBS) for 10 min and permeabilized with 0.5% Triton-X100 (15 min). The cover slips were incubated for 1 h with rabbit anti-H3 K27 trimethylated antibody (Millipore, Milan, Italy) and biotinylated anti-rabbit antibodies, and subsequently with 1% PFA/1 × PBS (10 min), 0.1 N HCl (8 min), 0.5% Triton-X100 (5 min), and 20% glycerol/1 × PBS (at least 1 h). Finally, after repeated freeze/thawing in liquid nitrogen, the cells were treated with pepsin solution (2.5 mg/ml pepsin in 0.01 N HCL at 37°C) for 5 min, and the cover slips were stored at 4°C in 50% formamide/2 × saline-sodium citrate (SSC).

### DNA probe labeling, hybridization and detection

The chromosome 4 painting probe was labeled by means of degenerated oligonucleotide primed (DOP)-PCR in the presence of tetramethyl-6-carboxyrhodamine-deoxyuridine triphosphate (TAMRA-dUTP). The BAC clone RP11-463J17 was labeled with Texas Red-dUTP, and the BAC containing D4Z4 repeats (derived from a human genomic library screening) (B. Bodega, unpublished results), was labeled with digoxigenin-dUTP by nick-end translation. Between 100 ng and 1 μg of each probe were mixed with a 10-fold excess of human Cot-1 DNA. *In situ *hybridization was performed for 48 h, followed by three 5-min stringency washes in 0.1 × SSC (60°C). The biotin-tagged H3K27me3 epitopes were detected by means of Avidin-Alexa488, the CH255-39M12 FISH probe sequentially with mouse anti-digoxigenin Cy5 and goat anti-mouse Cy5 antibodies. The 3D-fixed nuclei were counterstained for 5 min with 2 μg/ml DAPI.

### Laser scanning confocal microscopy and image processing

A Leica TCS SP5 laser scanning confocal microscope (Leica Microsystems, Wetzlar, Germany) with beam splitters tuned for DAPI, Alexa 488, TAMRA, TexasRed and Cy5 was used to scan the nuclei, with an axial distance of 200 nm between consecutive light optical sections yielding separate stacks of 8-bit grayscale images for each fluorochrome channel (pixel size 80 to 120 nm). The confocal image stacks were processed using ImageJ software [43].

### 3C Assay

The 3C assay was performed as previously described [34,35] with minor adaptations. A total of 5 × 10^7 ^cells were resuspended in 2 ml of 4°C cold cell lysis buffer (10 mM Tris, pH 8.0, 10 mM NaCl, 0.2% NP40 and protease inhibitors), and incubated for 1 h at 4°C, and the nuclei (1 × 10^7^) were crosslinked in a buffer containing formaldeyde 1% in 10 mM Tris-Cl, pH 7.9, 10 mM MgCl_2_, 50 mM NaCl and 1 mM dithiothreitol. The reaction was quenched by the addition of 0.125 M glycine. The crosslinked nuclei were resuspended in 500 μl of restriction enzyme buffer and, after the addition of 0.1% sodium dodecyl sulfate (SDS), incubated at 37°C for 1 h. Digestion was performed using 800 U of *Pvu*II at 37°C overnight. The reaction was diluted to a final concentration of 2.5 ng/μl in a ligation reaction buffer, and 4,000 U of T4 DNA ligase (NEB, Celbio, Milan, Italy) were added. The ligations were incubated at 4°C for 8 h. The samples were treated with proteinase K and incubated overnight at 65°C to reverse the formaldehyde crosslinks. The control templates were two BACs spanning the *FRG1 *region and the D4Z4 array (RP1-226K22 and a D4Z4-containing BAC isolated from a genomic library screening) (B. Bodega, unpublished results). Equimolar amounts of the BACs were mixed and digested with *Pvu*II, which was followed by ligation in 20 μl. The ligation was controlled as previously described [34]. The subsequent experiments made use of an amount of DNA that would amplify within the linear range. As a standard, the 5' side of each *Pvu*II fragment was used to design the primers, the sequences of which are available upon request. The PCR products were quantified using the Typhoon 9200 Image Quant program (GE Healthcare, Milan, Italy). A more detailed protocol is given in Additional file 5.

## Authors' contributions

BB conceived and designed this study, set up and performed ChIP and 3C experiments, statistical analyses, analyzed the data and wrote the manuscript; GDCR carried out 3C experiments; FG performed 3D FISH assay; SC performed expression and western blot analyses; SB analyzed the differentiation capability of myoblasts; MM collected samples and isolated myoblasts used in this study; RM and AM participated in critically reviewing the data; SM conceived and analyzed 3D FISH experiments and wrote the manuscript; EG and EB conceived the study, participated in its coordination, and wrote the manuscript. All authors read and approved the final manuscript.

## Supplementary Material

Additional file 1 **Myogenic differentiation properties of myoblasts derived from healthy donors and facioscapulohumeral muscular dystrophy (FSHD) patients**. **(a) **Human muscle cells utilized in this study: for each line specimen, sex and age at biopsies is indicated; for FSHD patients, D4Z4 contraction is reported. The index of fusion was calculated at days 4 and 8 of myogenic differentiation and reported. Human myoblast differentiation was monitored at 2, 4 and 8 days by **(b) **immunostaining for sarcomeric myosin (green) and MyoD (red) (nuclei are in blue as visualized by 4',6-diamidino-2-phenylindole (DAPI)), and **(c) **by reverse transcription polymerase chain reaction (RT-PCR) analysis of myosin, MyoD, and myogenin muscle-specific markers. 18S rRNA was used as control. **(d) **Expression of sarcomeric myosin was monitored at days 0, 1, 4, and 8 of myogenic differentiation by quantitative (q)RT-PCR relative to glyceraldehyde 3-phosphate dehydrogenase (GAPDH) expression for all cell lines utilized. Click here for file

Additional file 2 **Distribution on human chromosomes of sequences amplified in chromatin immunoprecipitation (ChIP) and methylated DNA immunoprecipitation (MeDIP) experiments**. The table shows the results obtained from a polymerase chain reaction (PCR) screening performed to test the chromosome specificity of the regions analyzed in the ChIP and MeDIP assays; the panel of human somatic cell hybrids were supplied by M. Rocchi (see main text). PCR primer pairs were derived from human chromosome 4 databank sequences [44], and are reported in Additional file 3. Click here for file

Additional file 3 **Primer pairs utilized in this study for chromatin immunoprecipitation (ChIP), methylated DNA immunoprecipitation (MeDIP) and reverse transcription polymerase chain reaction (RT-PCR) applications**. All the primer pairs utilized for sequencing and PCR-based analyses are listed in the table. For each primer, identification, 5' to 3' sequence and their application are reported. Click here for file

Additional file 4 **Facioscapulohumeral muscular dystrophy-related gene 1 (*FRG1*) promoter and D4Z4 units undergo to the same chromatin remodeling events during myoblasts differentiation**. **(a)**(i) A simplified scheme of the FRG1 promoter showing the position of one CarG box responsive element (sequence in red) and two E-boxes (sequences in green) in respect to the ATG and transcription start site (+1). Arrowheads indicate primer position for FRG1 A and B polymerase chain reaction (PCR) experiments (see Additional file 3 for primer sequences). (ii) Chromatin immunoprecipitation (ChIP) experiments were carried out on myoblasts and myotubes with antibodies anti-H3K27me3 (K27), anti-Ezh2 and anti-YY1. Input DNA (+) represents total chromatin, while the preimmune chromatin represents the immunoprecipitate obtained with a rabbit IgG. (iii) DNA methylation analysis by methylated DNA immunoprecipitation (MeDIP) assay on FRG1 A and FRG1 B regions of human myoblasts with antibody anti-5-methyl cytidine. Input DNA represents the supernatant from each genomic DNA. **(b)**(i) A simplified scheme of the D4Z4 unit showing the position of two CarG box responsive elements (sequences in red). Arrowheads indicate primer position for Lsau, D4Z4 binding element (DBE)1 and DBE2 subregions (see Additional file 3 for primers); (ii) DNA methylation analysis by MeDIP assay on DBE1 and Lsau D4Z4 subregions of human myoblasts with antibody anti-5-methyl cytidine. Input DNA represents the supernatant from each genomic DNA. (iii) DNA methylation analysis by MeDIP assay of D4Z4 repeats; MeDIP experiments were performed with antibody anti-5-methyl cytidine on somatic cell hybrids containing as single human representatives chromosome 4 (4), chromosome 10 (10), chromosome 15 (15) and chromosome 22 (22); the input DNA was an aliquot of the supernatant from each centrifuged genomic DNA. All DNA regions were PCR amplified on input and immunoprecipitated samples. Click here for file

Additional file 5 **Chromosome conformation capture (3C) detailed protocol for human myoblasts and myotubes**. The detailed chromosome conformation capture procedure applied on human myoblasts and myotubes is reported. The primers utilized in this experiment are also listed. Click here for file

Additional file 6 **Example of chromosome conformation capture (3C) analysis performed on myoblasts with D1 bait on D4Z4 unit**. **(a) **Amplification bands on 3C artificial control (bacterial artificial chromosomes (BACs) of 4q locus digested and ligated) and on 3C experiment performed on human myoblasts using D1 as bait and F series as prays. **(b) **Amplifications of F7-D1 and F18-D1 interactions on BACs, myoblasts and myotubes from three different experiments; on the right, amplification of facioscapulohumeral muscular dystrophy-related gene 1 (FRG1) promoter (with primers FRG1A) represents a loading control of the amount of 3C template used for the polymerase chain reaction (PCR) analysis on BACs, myoblasts and myotubes. **(c) **Quantification of the F7-D1 and F18-D1 interactions showed in **(b)**; band intensities were measured using a Typhoon 9200 phosphoscanner and Image Quant analysis software; PCR intensities were first normalized on the corresponding the loading control, and expressed as fold factor of the BAC PCR amplification; standard deviation of the mean is indicated. A two-tailed t test was applied for statistical analysis. Asterisks indicate the differences that are statistically relevant; F7-D1 myoblasts/F7-D1 myotubes, *P *= 0.024. Click here for file

Additional file 7 **Facioscapulohumeral muscular dystrophy-related gene 1 (*FRG1*) promoter and D4Z4 array physically interact within chromosome 4**. **(a) **Diagram of the genomic region analyzed in chromosome conformation capture (3C) experiments; *Pvu*II restriction sites are indicated (thin vertical lines); arrowheads indicate primer positions, F series in *FRG1 *gene and D series in D4Z4 repeats. Red rectangles indicate the location of CarG boxes. **(b) **Crosslinking frequencies between the fixed *Pvu*II fragment D1 (D4Z4 repeats) and the rest of the facioscapulohumeral muscular dystrophy (FSHD) locus in monochromosomal somatic cell hybrid retaining the human chromosome 4. All data points were generated from an average of three independent experiments. The standard error of the mean is indicated. One-way analysis of variance (ANOVA) was applied for statistical analysis; a = 0.05, *P *= 7.36*10^-5^. Primers are listed in Additional file 5, in the detailed 3C protocol section. **(c) **Crosslinking frequencies between the fixed *Pvu*II fragment C1 (DUX4c region) and the rest of the FSHD locus in myoblasts (black) and myotubes (pink). All data points were generated from an average of three independent experiments performed in control myoblasts cell lines. The standard error of the mean is indicated. One-way ANOVA was applied for statistical analysis; myoblasts: a = 0.05, *P *= 0.002; myotubes: a = 0.05, *P *= 0.061. Click here for file
