# Supplementary figures and images for: Remodeling of the chromatin structure of the facioscapulohumeral muscular dystrophy (FSHD) locus and upregulation of FSHD-related gene 1 (FRG1) expression during human myogenic differentiation
Source: BMC Biol. 2009 Jul 16;7:41. doi: 10.1186/1741-7007-7-41 (PMC2719609; doi:10.1186/1741-7007-7-41)

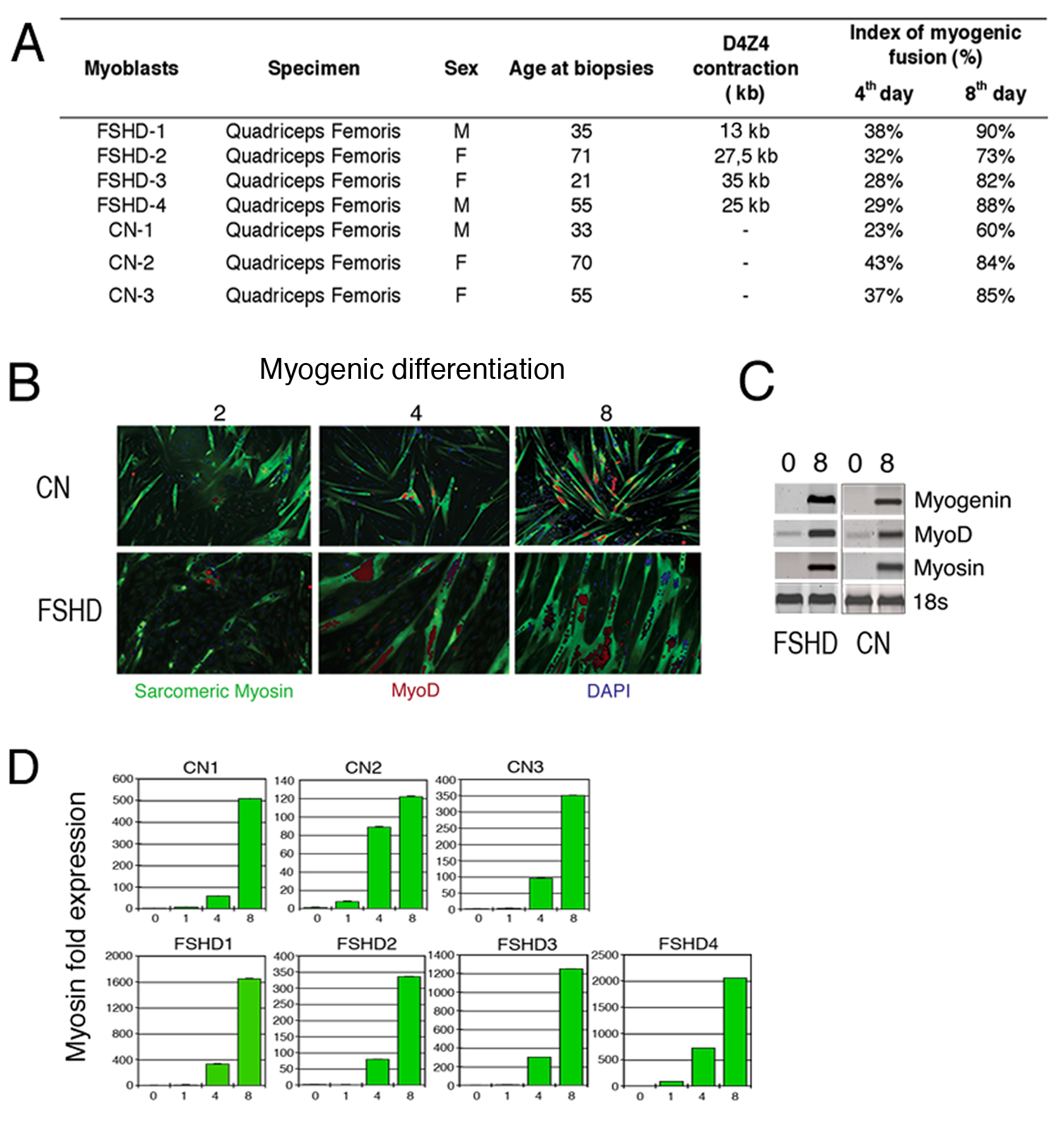

Supplement: Additional file 1 — Myogenic differentiation properties of myoblasts derived from healthy donors and facioscapulohumeral muscular dystrophy (FSHD) patients. (a) Human muscle cells utilized in this study: for each line specimen, sex and age at biopsies is indicated; for FSHD patients, D4Z4 contraction is reported. The index of fusion was calculated at days 4 and 8 of myogenic differentiation and reported. Human myoblast differentiation was monitored at 2, 4 and 8 days by (b) immunostaining for sarcomeric myosin (green) and MyoD (red) (nuclei are in blue as visualized by 4',6-diamidino-2-phenylindole (DAPI)), and (c) by reverse transcription polymerase chain reaction (RT-PCR) analysis of myosin, MyoD, and myogenin muscle-specific markers. 18S rRNA was used as control. (d) Expression of sarcomeric myosin was monitored at days 0, 1, 4, and 8 of myogenic differentiation by quantitative (q)RT-PCR relative to glyceraldehyde 3-phosphate dehydrogenase (GAPDH) expression for all cell lines utilized. [file 1741-7007-7-41-S1.tiff]

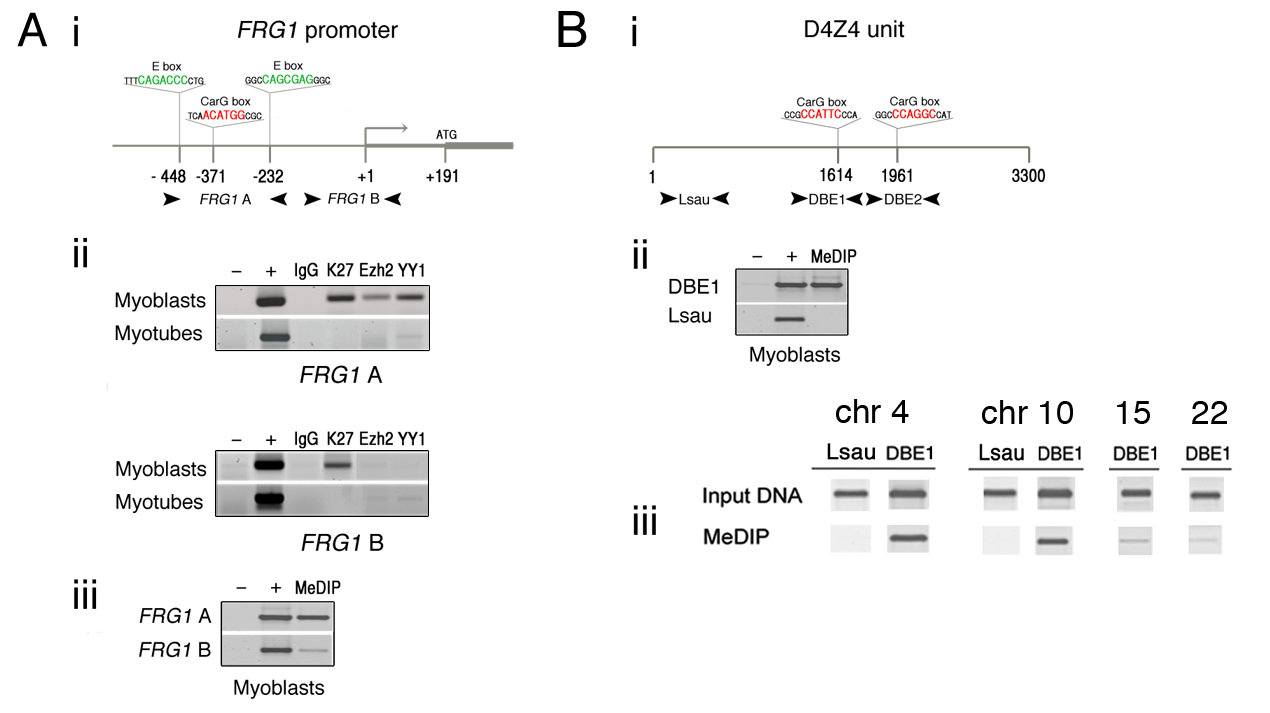

Supplement: Additional file 4 — Facioscapulohumeral muscular dystrophy-related gene 1 (FRG1) promoter and D4Z4 units undergo to the same chromatin remodeling events during myoblasts differentiation. (a)(i) A simplified scheme of the FRG1 promoter showing the position of one CarG box responsive element (sequence in red) and two E-boxes (sequences in green) in respect to the ATG and transcription start site (+1). Arrowheads indicate primer position for FRG1 A and B polymerase chain reaction (PCR) experiments (see Additional file 3 for primer sequences). (ii) Chromatin immunoprecipitation (ChIP) experiments were carried out on myoblasts and myotubes with antibodies anti-H3K27me3 (K27), anti-Ezh2 and anti-YY1. Input DNA (+) represents total chromatin, while the preimmune chromatin represents the immunoprecipitate obtained with a rabbit IgG. (iii) DNA methylation analysis by methylated DNA immunoprecipitation (MeDIP) assay on FRG1 A and FRG1 B regions of human myoblasts with antibody anti-5-methyl cytidine. Input DNA represents the supernatant from each genomic DNA. (b)(i) A simplified scheme of the D4Z4 unit showing the position of two CarG box responsive elements (sequences in red). Arrowheads indicate primer position for Lsau, D4Z4 binding element (DBE)1 and DBE2 subregions (see Additional file 3 for primers); (ii) DNA methylation analysis by MeDIP assay on DBE1 and Lsau D4Z4 subregions of human myoblasts with antibody anti-5-methyl cytidine. Input DNA represents the supernatant from each genomic DNA. (iii) DNA methylation analysis by MeDIP assay of D4Z4 repeats; MeDIP experiments were performed with antibody anti-5-methyl cytidine on somatic cell hybrids containing as single human representatives chromosome 4 (4), chromosome 10 (10), chromosome 15 (15) and chromosome 22 (22); the input DNA was an aliquot of the supernatant from each centrifuged genomic DNA. All DNA regions were PCR amplified on input and immunoprecipitated samples. [file 1741-7007-7-41-S4.tiff]

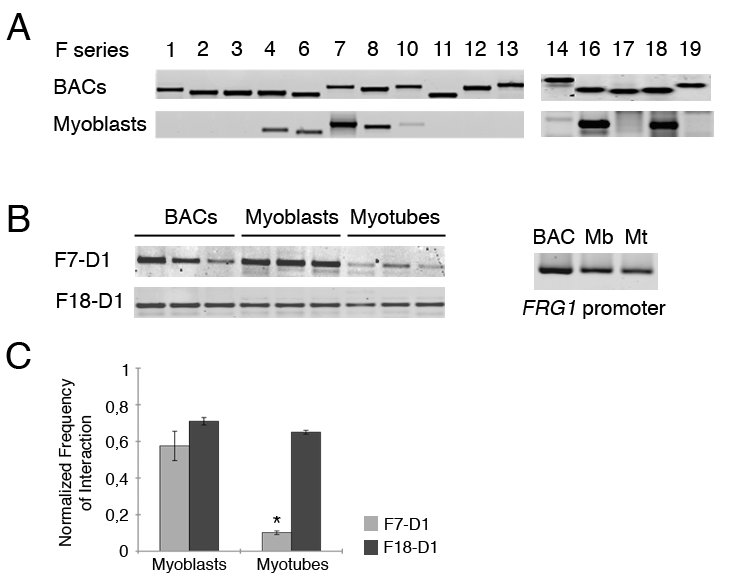

Supplement: Additional file 6 — Example of chromosome conformation capture (3C) analysis performed on myoblasts with D1 bait on D4Z4 unit. (a) Amplification bands on 3C artificial control (bacterial artificial chromosomes (BACs) of 4q locus digested and ligated) and on 3C experiment performed on human myoblasts using D1 as bait and F series as prays. (b) Amplifications of F7-D1 and F18-D1 interactions on BACs, myoblasts and myotubes from three different experiments; on the right, amplification of facioscapulohumeral muscular dystrophy-related gene 1 (FRG1) promoter (with primers FRG1A) represents a loading control of the amount of 3C template used for the polymerase chain reaction (PCR) analysis on BACs, myoblasts and myotubes. (c) Quantification of the F7-D1 and F18-D1 interactions showed in (b); band intensities were measured using a Typhoon 9200 phosphoscanner and Image Quant analysis software; PCR intensities were first normalized on the corresponding the loading control, and expressed as fold factor of the BAC PCR amplification; standard deviation of the mean is indicated. A two-tailed t test was applied for statistical analysis. Asterisks indicate the differences that are statistically relevant; F7-D1 myoblasts/F7-D1 myotubes, P = 0.024. [file 1741-7007-7-41-S6.tiff]

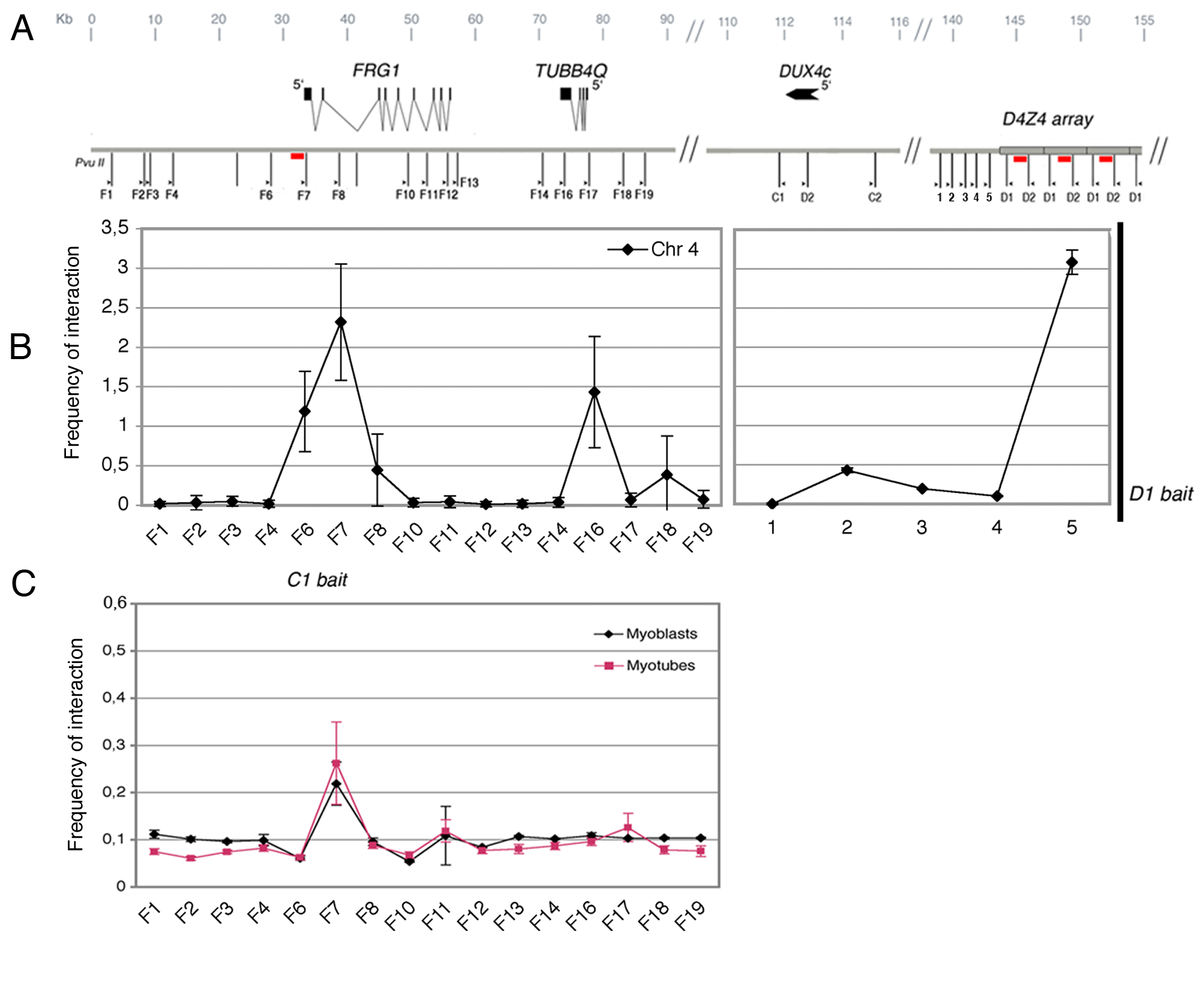

Supplement: Additional file 7 — Facioscapulohumeral muscular dystrophy-related gene 1 (FRG1) promoter and D4Z4 array physically interact within chromosome 4. (a) Diagram of the genomic region analyzed in chromosome conformation capture (3C) experiments; PvuII restriction sites are indicated (thin vertical lines); arrowheads indicate primer positions, F series in FRG1 gene and D series in D4Z4 repeats. Red rectangles indicate the location of CarG boxes. (b) Crosslinking frequencies between the fixed PvuII fragment D1 (D4Z4 repeats) and the rest of the facioscapulohumeral muscular dystrophy (FSHD) locus in monochromosomal somatic cell hybrid retaining the human chromosome 4. All data points were generated from an average of three independent experiments. The standard error of the mean is indicated. One-way analysis of variance (ANOVA) was applied for statistical analysis; a = 0.05, P = 7.36*10-5. Primers are listed in Additional file 5, in the detailed 3C protocol section. (c) Crosslinking frequencies between the fixed PvuII fragment C1 (DUX4c region) and the rest of the FSHD locus in myoblasts (black) and myotubes (pink). All data points were generated from an average of three independent experiments performed in control myoblasts cell lines. The standard error of the mean is indicated. One-way ANOVA was applied for statistical analysis; myoblasts: a = 0.05, P = 0.002; myotubes: a = 0.05, P = 0.061. [file 1741-7007-7-41-S7.tiff]
