# Supplementary material for: Remodeling of the chromatin structure of the facioscapulohumeral muscular dystrophy (FSHD) locus and upregulation of FSHD-related gene 1 (FRG1) expression during human myogenic differentiation
Source: BMC Biol. 2009 Jul 16;7:41. doi: 10.1186/1741-7007-7-41 (PMC2719609; doi:10.1186/1741-7007-7-41)
Supplement: Additional file 2 — Distribution on human chromosomes of sequences amplified in chromatin immunoprecipitation (ChIP) and methylated DNA immunoprecipitation (MeDIP) experiments. The table shows the results obtained from a polymerase chain reaction (PCR) screening performed to test the chromosome specificity of the regions analyzed in the ChIP and MeDIP assays; the panel of human somatic cell hybrids were supplied by M. Rocchi (see main text). PCR primer pairs were derived from human chromosome 4 databank sequences [44], and are reported in Additional file 3. [file 1741-7007-7-41-S2.doc]

Supplementary Table I – Distribution on human chromosomes of sequences amplified in ChiP and MeDIP experiments

| **Human**  **Chromosomes** | **1** | **2** | **3** | **4** | **5** | **6** | **7** | **8** | **9** | **10** | **11** | **12** | **13** | **14** | **15** | **16** | **17** | **18** | **19** | **20** | **21** | **22** | **X** | **Y** |
| --- | --- | --- | --- | --- | --- | --- | --- | --- | --- | --- | --- | --- | --- | --- | --- | --- | --- | --- | --- | --- | --- | --- | --- | --- |
| **FRG1 A** | - | - | - | + | - | - | - | - | - | - | - | - | - | - | - | - | - | - | - | - | - | - | - | - |
| **FRG1 B** | - | - | - | + | - | - | - | - | - | - | - | - | - | - | - | - | - | - | - | - | - | - | - | - |
| **Lsau** | - | - | - | + | - | - | - | - | - | + | - | - | - | - | - | - | - | - | - | - | - | - | - | - |
| **DBE1** | - | - | + | + | - | - | - | - | + | + | - | - | + | + | + | - | - | - | - | - | + | + | + | + |
| **DBE2** | - | - | + | + | - | - | - | - | + | + | - | - | + | + | + | - | - | - | - | - | + | + | + | + |
