# Supplementary material for: Remodeling of the chromatin structure of the facioscapulohumeral muscular dystrophy (FSHD) locus and upregulation of FSHD-related gene 1 (FRG1) expression during human myogenic differentiation
Source: BMC Biol. 2009 Jul 16;7:41. doi: 10.1186/1741-7007-7-41 (PMC2719609; doi:10.1186/1741-7007-7-41)
Supplement: Additional file 3 — Primer pairs utilized in this study for chromatin immunoprecipitation (ChIP), methylated DNA immunoprecipitation (MeDIP) and reverse transcription polymerase chain reaction (RT-PCR) applications. All the primer pairs utilized for sequencing and PCR-based analyses are listed in the table. For each primer, identification, 5' to 3' sequence and their application are reported. [file 1741-7007-7-41-S3.doc]

**Supplementary Table 2 – Primer pairs and experimental application**

| **Primer pair** | **Sequence** | **Application** |
| --- | --- | --- |
| *FRG1* B F | TCTACAGAGACGTAGGCTGTCA | ChIP/MeDIP |
| *FRG1* B R | CTTGAGCACGAGCTTGGTAG | ChIP/MeDIP |
| *FRG1* A F | AGCTTCCTCCCATCTCTGTCG | ChIP/MeDIP |
| *FRG1* A R | GTGTGTGCAGGAAACAAGCA | ChIP/MeDIP |
| *G6PD F* | TAGGGCCGCATCCCGCTCCGGAGAGAAGTCT | ChIP |
| *G6PD R* | CTGCCATACCCGCTGCCGCTGCTCTGCATC | ChIP |
| *FRG1* ex1 F | GCCCCGACTCACATACTCGT | RT PCR – qRT – PCR |
| *FRG1* ex2 R | TCAAGCTGGGTTTCTTCATCTTC | RT PCR – qRT – PCR |
| *FRG1* 1F^a^ | TCTACAGAGACGTAGGCTGTCA | qRT – PCR |
| *FRG1* Rb^a^ | CTTGAGCACGAGCTTGGTAG | qRT – PCR |
| 18s subunit F | CCGATTGGATGGTTTAGTGAGG | RT PCR – qRT-PCR |
| 18s subunit R | GATCCTTCCGCAGGTTCAC | RT PCR – qRT-PCR |
| beta 2 M F | TGAGTATGCCTGCCGTGTGA | qRT – PCR |
| beta 2 M R | GGCATCTTCAAACCTCCATGA | qRT – PCR |
| *GAPDH*-RT F | TGCACCACCAACTGCTTAGC | qRT – PCR |
| *GAPDH*-RT R | GGCATGGACTGTGGTCATGAG | qRT – PCR |
| MYH2 F | GGACCAACTGAGTGAACTGAAA | RT PCR – qRT-PCR |
| MYH2 R | TTGCCTCTTGATAACTGAGACAC | RT PCR – qRT-PCR |
| MyoD F | CATCCGCTACATCGAAGGTC | RT PCR |
| MyoD R | TAGTAGGCGGTGTCGTAGCC | RT PCR |
| MyoG F | TCAACCAGGAGGAGCGTGAC | RT PCR |
| MyoG R | TGTAGGGTCAGCCGTGAGCA | RT PCR |
| Lsau F | AGCCGCCTGGGCTGTGGGAGC | ChIP/MeDIP |
| Lsau R | TCTGCGTTCCGCCGCCAGC | ChIP/MeDIP |
| DBE 1 F^b^ | AGGCCTCGACGCCCTGGGTC | ChIP/MeDIP |
| DBE 1 R^b^ | TCAGCCGGACTGTGCACTGCGGC | ChIP/MeDIP |
| DBE 2 F | ACGGAGACTCGTTTGGAC | ChIP/MeDIP |
| DBE 2 R | TGGAAAGCGATCCTTCTC | ChIP/MeDIP |

a: van Deutekom JC et al. 1996 b: Gabellini D et al., 2002
