# Supplementary material for: Remodeling of the chromatin structure of the facioscapulohumeral muscular dystrophy (FSHD) locus and upregulation of FSHD-related gene 1 (FRG1) expression during human myogenic differentiation
Source: BMC Biol. 2009 Jul 16;7:41. doi: 10.1186/1741-7007-7-41 (PMC2719609; doi:10.1186/1741-7007-7-41)
Supplement: Additional file 5 — Chromosome conformation capture (3C) detailed protocol for human myoblasts and myotubes. The detailed chromosome conformation capture procedure applied on human myoblasts and myotubes is reported. The primers utilized in this experiment are also listed. [file 1741-7007-7-41-S5.doc]

**3C detailed protocol for human myoblasts and myotubes**

For 3C analysis, the *PvuII* restriction enzyme at high concentration (50U/ml, Neb, R0151M) was used. Possibility of star activity was previously checked on plasmid digestion, and it was negative. For myoblasts and myotubes, a total of 50*10^7^ cells were resuspended in 2 ml of 4°C cold cell lysis buffer (10 mM KCl, 10 mM Tris pH 8.0, 0,5% NP-40, in the presence of protease inhibitors) and incubated with rotation at 4°C for at least one hour. Lysis was checked at microscope, and eventually lysis was completed with ten strokes using a Dounce homogenizer (pestle A). Nuclei (about 25 – 35 * 10^7^) were pelletted and resuspended in 5 ml of crosslinking buffer (10 mM Tris pH 8, 10 mM MgCl_2_, 50 mM NaCl, 1mM DTT in the presence of protease inhibitors) and crosslinked with 1% formaldehyde for 10 minutes at room temperature. Then, the reaction was quenched by the addition of glycine to a final concentration of 0,125 M. Crosslinked nuclei were washed two times with PBS and then resuspended in 500 ml of restriction enzyme Buffer Neb2 complemented with SDS to the final concentration of 0.3%, and incubated at 37°C for 1 hour. Triton X-100 was then added to the final concentration of 1.8% to sequester SDS and incubated again for 1 hour at 37°C. Before adding the restriction enzyme, 5 ml aliquot of the sample was collected and labelled as undigested genomic DNA control (UND), necessary for the calculation of efficiency of digestion. Digestion was performed with 600 - 800 U of restriction enzyme at 37 °C overnight with constant agitation. The next day, the restriction enzyme was inactivated by the addition of SDS to 1.6% and incubation at 65 °C for 20 minutes. At this step, another aliquot of 5 ml of the sample was collected and labelled as digested genomic DNA control (D), for the determination of digestion and crosslinking efficiency (see below). The reaction was diluted into 6 ml ligation reaction buffer containing 1% Triton X-100, 50 mM Tris pH 7.5, 10 mM MgCl_2_, 10 mM DTT, 0.1 mg/ml bovine serum albumin, 1 mM ATP and incubated 1 hour with constant agitation at 37°C. Then, 4000 U of T4 DNA Ligase (Neb) were added and ligations were performed at 4°C for 8 hours. EDTA (to a concentration of 10 mM) was added to stop the reactions. Samples were treated with 500 μg of Proteinase K and incubated overnight at 65 °C to reverse the formaldehyde crosslinks.

The following day, the DNA was purified by phenol extraction and isopropanol precipitation. Samples were redissolved in 150 ml of deionized water and stored at -20°C for further applications.

As control templates, we used two BACs spanning the *FRG1* region and the D4Z4 array (RP1-226K22 and a D4Z4 containing BAC isolated from a genomic library screening (Bodega, unpublished results). The DNA from each BAC was quantified by realtime PCR, using primers that anneal in the backbone. Equimolar amounts of different BACs were mixed and digested with *PvuII*, followed by ligation in 20 μl. The mix was purified by phenol extraction and ethanol precipitation. An appropriate amount of DNA that would amplify within the linear range was subsequently used for the experiments (50 ng).

Biological parameters, such as the heterogeneity of the cells, also have to be considered. Thus, when comparing two different cell types, we normalize to the total amount of DNA in the reaction, also considering the efficiency of digestion and ligation; this allows compensation of the varying efficiencies of the application due to the different source material. The efficiency of digestion was quantified by real-time PCR, amplifying a fragment spanning the F7, F16 and F18 PvuII site in UND and D samples and a FRG1 B promoter region as loading control, to normalize the results of the qPCR; the Ct values (cycle thresholds) were used to calculate the restriction efficiency according to the formula reported in Hagege et al. 2007: % restriction = 100 – 100/2^(Ct_D_- Ct_UND_); we further process only the 3C samples were efficiently digested (>70%). To control the efficiency of ligation, a linear *EcoR*I-digested plasmid was added to all the preparations before ligation. The ligated plasmid was quantified by real-time PCR, amplifying a fragment spanning the *EcoR*I site. To compare the frequency of interactions in different cell types, cross-linking frequencies of specific sites, F7, F16 and F18 were determined by quantification of PCR products from cross-linked templates (3C samples ligated at low concentrations) and control samples (BAC samples ligated at high concentrations). These interactions were used for normalization; cross-linking frequency (X) was calculated as a ratio of the amount of the mean of F7, F16 and F18 PCR products obtained from 3C samples (A) to the amount of the mean of F7, F16 and F18 PCR products obtained on BAC samples (B) (Eivazova et al., 2004). These values were used to normalize the frequency of interactions between D1 and F7, F16 and F18 respectively, in different cell types (i.e. myoblasts, myotubes, FSHD, CN cell lines).

An appropriate amount of DNA that would amplify within the linear range was subsequently used for the experiments. A total of 33 rounds of PCR amplification were used. As standard, the 5' side of each restriction fragment was used to design primers. PCR products were run on 2% agarose gels, PCR products were quantified with the Typhoon 9200 Image Quant program (Amersham).

For measuring differences in the F7-D1 interactions between FSHD and controls, we used a Taq-Man based approach, as reported in Hagege et al., 2007. In this case, for normalizing the results we used a second TaqMan probe designed on the promoter of beta 2 microbulin gene, avoiding PvuII restriction sites.

Primer set:

F series:

F1: 5’ TCTGATGCTGTTGGATGGAA 3’

F2: 5’ TTAACTCAGAAGGGCCTGGA 3’

F3: 5’ GGATCCACTGAATTTGTGCAG 3’

F4: 5’ GGATGCTATTATTATCCCCCTTTT 3’

F6: 5’ TCCATGGATGTGCTCCAATA 3’

F7: 5’ CTCTAGCAGGAGGCGGTTC 3’

F8: 5’ TGGAAAGTGCTGTCTCCTTTC 3’

F10: 5’ CGAGCATGTCTGTAGGCAAA 3’

F11: 5’ AGACAGCCTCTGACCCTCCT 3’

F12: 5’ GCCGCTCATTCAAGTCGTAT 3’

F13: 5’ CCTGCCTGCCTCTGTAGACT 3’

F14: 5’ CCTCTGCATGTTGTGTGCTT 3’

F16: 5’ CATGTTCATGGCCAGCTTC 3’

F17: 5’ CCTCCCAAAGTGCTGGATTA 3’

F18: 5’ ACTTCCCAGAGAGGGCAAAC 3’

F19: 5’ TTGCAAATGGCAGACTCTCA 3’

D baits:

D1: 5’ ctccctcctaacgtcccttc 3’

D2: 5’ ggaaagcgatccttctcaaa 3’

C baits:

C1: 5’ tgaacctctgactcggtatgg 3’

C2: 5’ GCTGGCAGATCACCTGAGTT 3’

Primers for determination of digestion and crosslinking efficency:

F7 – PvuII F: 5’ CTCAGGGGCTCCTTGAGAG 3’

F7 – PvuII R: 5’ GTTTCTGCCGGGGAGCTA 3’

F16 – PvuII F: 5’ CATGTTCATGGCCAGCTTC 3’

F16 – PvuII R: 5’ GCTACCATGAGTGGGGTCAC 3’

F18 – PvuII F: 5’ ACTTCCCAGAGAGGGCAAAC 3’

F18 – PvuII R: 5’ CACGTGAGCTTCACCAGAAC 3’

Primers used for normalization as loading control:

FRG1 B F: 5’ TCTACAGAGACGTAGGCTGTCA 3’

FRG1 B R: 5’ CTTGAGCACGAGCTTGGTAG 3’

Taq-Man probe for F7-D1 interaction: 5’ AATCCTCAGGGGCTCCTTGAGAG 3’

Taq-Man probe for beta2M promoter: 5’ CCACCACCACGAAATGGCGGCACC 3’

Beta 2M forward: 5’ CCACTTCCTCTTCTCACTGTTCC 3’

Beta 2M reverse: 5’ ACATGACAGGCAGACCCATTAAG 3’.
